# Positive impacts of typical desert photovoltaic scenarios in China on the growth and physiology of sand-adapted plants

**DOI:** 10.3389/fpls.2024.1515896

**Published:** 2025-01-20

**Authors:** Ruibing Meng, Zhongju Meng, Xiaomeng Ren, Jiale Cai, Xufang Tong

**Affiliations:** ^1^ College of Desert Control and Engineering, Inner Mongolia Agricultural University, Hohhot, China; ^2^ Key Laboratory of Aeolian Physics and Desertification Control Engineering from Inner Mongolia Autonomous Region, Inner Mongolia Agricultural University, Hohhot, China; ^3^ Key Laboratory of Desert Ecosystem Conservation and Restoration, State Forestry and Grassland Administration of China, Inner Mongolia Agricultural University, Hohhot, China; ^4^ Inner Mongolia Institute of Meteorological Sciences, Hohhot, China

**Keywords:** desert, plant physiology, photovoltaic, vegetation restoration, growth characteristics

## Abstract

Desert regions are characterized by complex terrain, frequent wind–sand activities, and extreme environmental conditions making vegetation recovery after disturbances difficult. The construction of large-scale photovoltaic (PV) power stations presents a significant challenge in balancing with vegetation protection. This study focused on a large PV site in the Hobq Desert examining the growth characteristics of *Astragalus adsurgens* at different positions within fixed PV arrays. It also analyzed changes in chlorophyll (Chl), soluble sugar (SS), soluble protein (SP), enzyme activity, and other physiological indicators to evaluate the plant’s adaptive mechanisms to the presence of PV panels. The results showed that *A. adsurgens* near the PV panels exhibited significant growth advantages, with plant height, leaf length, and stem–leaf nutrient content greater than the those of the control (CK) showing an adaptive trend of elongation, thinning, and enlargement. During the growing season, *A. adsurgens* located before, behind, and under the panels increased Chl with environmental changes. The plants also adjusted their SS, SP, and other internal substance levels depending on their location relative to the panels. Notably, superoxide dismutase (SOD) and peroxidase (POD) activities were higher in all treated plants compared to those of CK, effectively removing O^2−^ and providing H_2_O_2_ protection, thereby delaying plant senescence and demonstrating strong adaptability. Through membership function analysis, the plant’s tolerance levels at various positions around the PV panels ranked under panels > before panels > behind panels > CK. In conclusion, *A. adsurgens* demonstrated adaptability to environmental changes at PV power stations by modifying its growth characteristics and physiological traits. These findings provide scientific evidence for the ecological industrial use of PV power stations in desert regions and offer practical guidance for vegetation restoration and ecological construction around such stations.

## 1 Introduction

With the depletion of fossil energy sources, like oil, natural gas, and coal, the global energy crisis has become a pressing challenge (Erdinc and Uzunoglu, 2012; Kanwal et al., 2022). In response, renewable, low-carbon, and sustainable energy sources are being actively pursued (Orr, 2016; Razi and Dincer, 2022). Solar energy, as a clean, abundant, and environmentally friendly resource, has become an ideal alternative to traditional fossil fuels (Sansaniwal et al., 2018; Lye et al., 2023). China, with its advanced photovoltaic (PV) technology, has taken a leading position in the global new energy sector, with its installed PV capacity reaching 6.09 trillion kW by 2023 (Binz et al., 2017; Pourasl et al., 2023). The rapid expansion of PV power plants not only enhances energy security but also supports green economic transformation and provides innovative solutions for land use and ecological protection (Zhang et al., 2023).

When selecting sites for PV power stations, the vast deserts and arid areas of northwest China offer ideal construction conditions for the PV industry (Huang et al., 2018; Chen et al., 2024). However, the unique and fragile ecosystems in desert regions make these areas both a focus of global environmental governance and a challenge for sustainable development (Cai et al., 2024). The natural environment in desert regions is harsh, with extremely fragile ecosystems and severe soil erosion (Chang et al., 2020). The rapid development of the PV industry has introduced new pressures on these vulnerable areas, especially during construction, where disturbances to the surface have exacerbated soil erosion (Yue et al., 2021a). Large-scale PV construction, covering multiple regions and involving high levels of mechanized operations, causes significant ecological damage through activities like excavation, filling, piling, and compression of the land (Randle-Boggis et al., 2020; Ren et al., 2022). Moreover, the tilt angle and layout of PV panels can accelerate wind erosion, further worsening the ecological environment (Wang et al., 2021).

In this context, implementing ecological restoration measures and vegetation reconstruction to control desertification has become urgent. The emerging “PV + desertification control” model, which combines forestry with PV systems in a complementary ecological management approach, offers an innovative pathway to developing renewable energy while restoring the ecosystem in desert regions (Rodriguez-Pastor et al., 2023). Through scientific planning, PV stations enhance solar energy efficiency and utilize the shading and moisture-enhancing effects of PV panels to promote vegetation growth (Sinke, 2019; Ezzaeri et al., 2020). This has improved land productivity, ecological restoration, and significant economic and social benefits (Liu et al., 2020). However, large-scale PV station construction affects local microclimates and plants, animals, and microorganisms’ growth, activity, and life cycles to varying degrees potentially altering the ecosystem’s carbon sequestration capacity (El Chaar et al., 2011; Javed et al., 2020). This issue has drawn widespread academic attention, with research covering topics such as changes in surface solar radiation, air temperature and humidity, and wind speed and direction. For instance, Li et al. (2023a) evaluated the environmental impacts of PV arrays in desert and lake regions; Yue et al. (2021b) analyzed soil moisture conditions in PV-covered areas and their buffer zones; Tang et al. (2021) studied the interference of PV arrays on wind and sand movement in the Hobq Desert; and Zhai et al. (2018) analyzed the stability of vegetation communities in PV power stations. These studies highlight that while the shading effect of PV panels creates a milder and more humid microclimate, the uneven sunlight distribution leads to varied plant growth affecting their physiological characteristics and adaptability. Therefore, further research is essential to explore the specific impacts of PV panels on plant physiology and ecology at different positions. Such studies can guide the optimization of ecological design in PV stations, enhance ecological restoration efficiency, and ensure sustainable environment development.

*A. adsurgens*, a perennial legume, known for its drought tolerance, cold resistance, robust root system, and rapid growth, has great potential for windbreak and sand fixation (Li et al., 2023b). Moreover, its nitrogen-fixing ability improves soil fertility, while the nutrient-rich stems and leaves can be used as valuable livestock feed (Han et al., 2024). These characteristics make it an ideal species to address the challenges of both ecological degradation and sustainable land use in PV plant areas. Therefore, this study selected a fixed PV station on the northern edge of the Hobq Desert as the research site, adopting an “electricity generation above, planting below” model to accelerate vegetation restoration by planting *A. adsurgens* in the PV station areas. The study aimed to investigate the growth suitability and planting potential of *A. adsurgens* in different positions of PV panels. By comparing the growth morphology and physiological indexes of *A. adsurgens* in different locations (before panels, behind panels, and under panels), and using the areas without PV panels as a control (CK), the impacts of PV panels on the growth of *A. adsurgens* was revealed. This study aims to evaluate the growth characteristics, physiological responses, and planting potential of *A. adsurgens* under different PV panel positions, providing insights into ecological restoration in desert PV stations. The research goals include the following: (1) quantifying the growth characteristics and changes in stem and leaf nutrient content of *A. adsurgens* in different PV panel positions; (2) exploring the adaptive strategies and physiological regulation mechanisms of *A. adsurgens* in different PV panel positions; (3) assess the potential for planting *A. adsurgens* at different locations within a PV station and propose control measures.

## 2 Materials and methods

### 2.1 Site description

**Figure 1:**
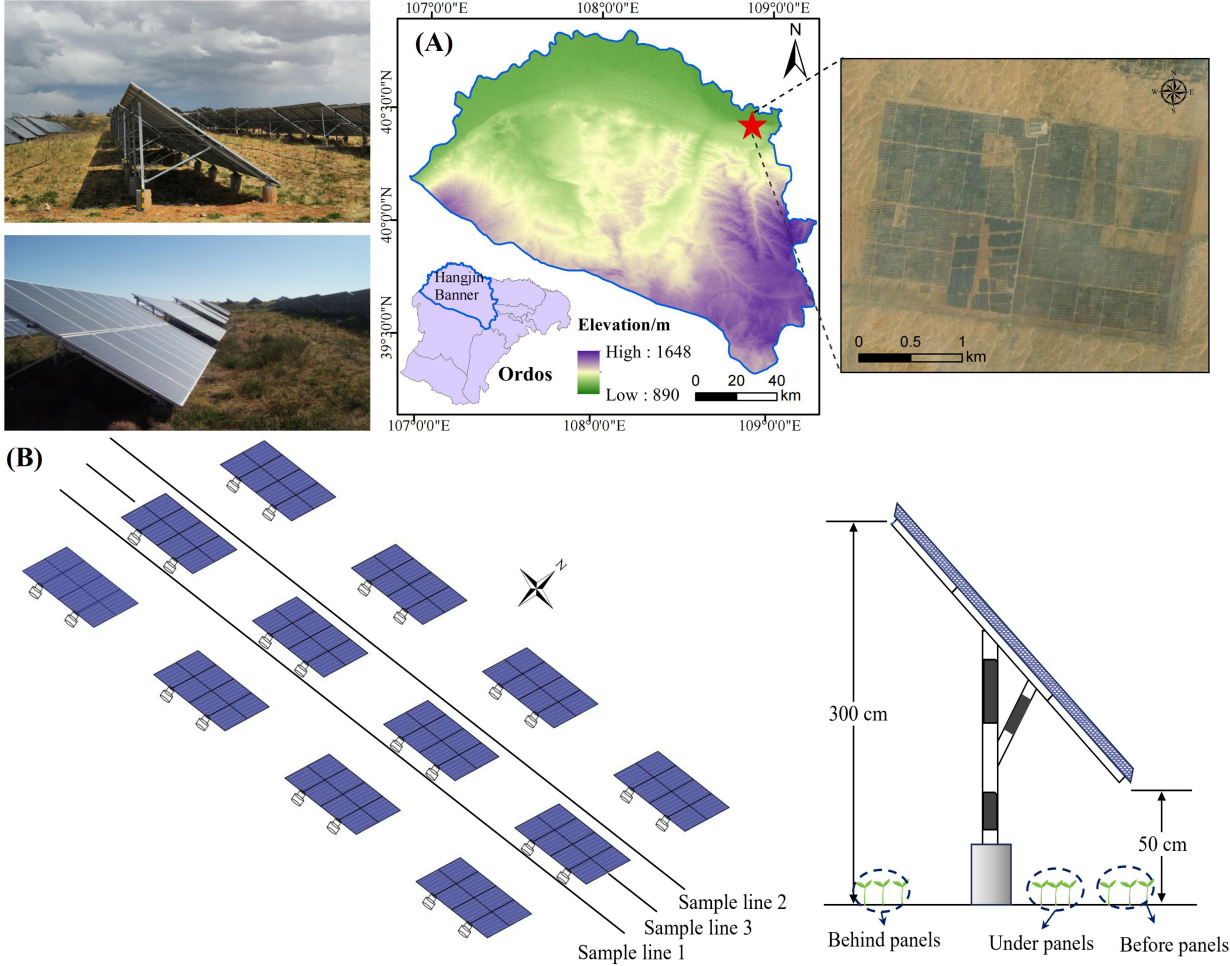
Comprehensive overview of the study area. **(A)** Geographical positioning map of the study region. **(B)** Schematic representation of sample line deployment and sampling sites within the study area.

The study area is located at the northern edge of the Hobq Desert, within the Duguitala Industrial Park in Hangjin Banner, Ordos City (107°10′ E–111°45′ E, 37°20′ N–39°50′ N) (**Figure 1A**). The region has a temperate continental monsoon climate, with an average annual temperature of 6°–7°C, low precipitation (average annual rainfall of 227 mm), and high evaporation (2,400 mm annually). The desert’s harsh conditions reflect the challenges posed by desertification and land degradation. The soil is primarily sandy, with an average vegetation coverage of less than 5%. The total annual solar radiation in this area is 597.9 KJ/cm² further underscoring its suitability for PV installations aimed at exploring sustainable energy solutions while addressing desertification (Tang et al., 2021).

The experimental plots are located within the third phase of the Yili Ecological PV Zone (100 MW), a fixed PV power station that began operating in 2016. The PV panels are arranged in a fixed east–west direction with a southward tilt of 36°, leaving a 900-cm north–south spacing and 110-cm east–west row spacing between arrays. Each row consists of 12 panels, and each panel contains 34 cells. The dimensions of each panel are 400 cm by 800 cm. The front edge of the panels is 50 cm above the ground, while the rear edge is 300 cm above the ground. The entire PV station is uniformly covered with a red clay substrate.

### 2.2 Experimental design

The experiment was conducted in the third phase of the Yili Ecological PV zone, within a fixed PV power station. This area serves as a specialized experimental zone for vegetation restoration. To optimize the use of space around the solar panels and enhance wind erosion control, *A. adsurgens* (a sand-binding plant) was planted in 2020 before, behind, and under the PV panels using hole-sowing methods. Given the underlying surface effects and to improve the survival rate of the plants, the area around the panels was leveled and treated with a uniform layer of red loam to ensure consistency in the substrate. The seeds were sown in holes during the post-wind season and before the onset of the rainy season, with a spacing of 0.6 m × 1 m between the plants and covered with 1–2 cm of soil.

**Figure 2:**
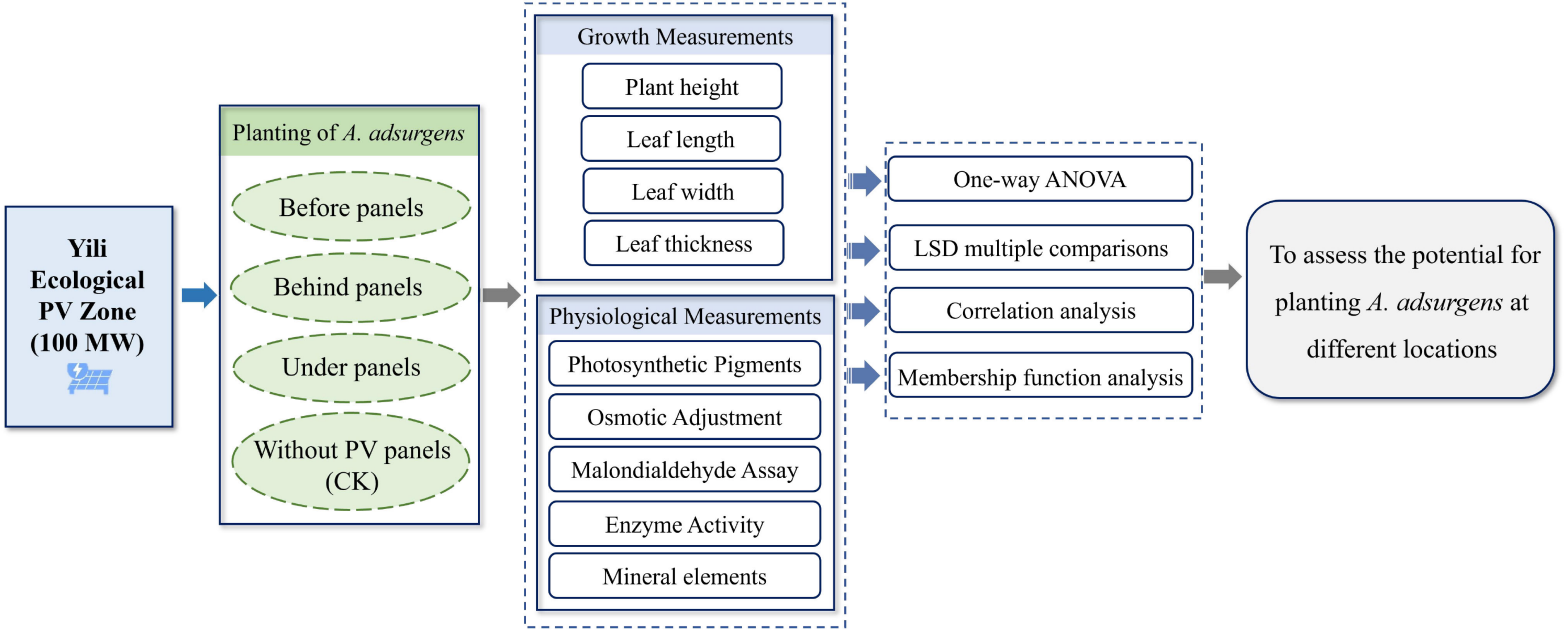
Flowchart of experimental method.

To investigate the plant’s growth characteristics and physiological responses, the 2022 growing season (June to August) was selected for the study. Three parallel rows of PV panels from the central region of the PV array were designated as the study area, with CK samples taken from the area outside the PV station without any panels. Three 50-m-long sample lines (sample lines 1, 2, and 3) were laid out in the east–west direction before, behind, and under the panels (**Figure 1B**). Specifically, plants before panels are located in open areas and exposed to direct sunlight (Monthly average 1 hour 38 minutes), while plants under panels are partially shaded and receive less sunlight by the location of the panels (Monthly average 10 hour 33 minutes). Plants behind panels were in shade for most of the day and received limited direct sunlight (Monthly average 9 hour 42 minutes). Sampling quadrats (50 cm × 50 cm) were established along each transect at 10-m intervals. Within each quadrat, 12 morphologically similar and evenly growing *A. adsurgens* plants were selected, and growth parameters such as plant height, leaf length, leaf width, and leaf thickness were measured monthly. To capture physiological responses, leaf samples from three plants per quadrat were collected and immediately placed in resealable bags and stored in a refrigerated cooler for transport to the laboratory. In the lab, leaves were separated from the midribs, and the appropriate sample mass for each physiological indicator was measured according to the experimental requirements. Field measurements were taken from the 24th to the 28th of each month, and laboratory analyses were completed within 7 days of sample collection. The specific method is shown in **Figure 2**.

### 2.3 Observation of shade duration

The artificial squatting timing method was used to observe and record the shading conditions of the before, behind, and under panels from 07:00 to 19:00 hours as the observation period under clear and cloudless weather conditions. Finally, the average value was found based on the shading time of each location in 3 months. According to the observation of the shade time of each position of the PV panels in the experimental sample site, the shade time of the before panels, behind panels, and under panels in June was 2 h 35 min, 9 h 10 min, and 10 h 20 min, respectively; in July, it was 2 h, 9 h 30 min, and 10 h 40 min, respectively; and in August, it was 20 min, 10 h 25 min, and 10 h 40 min, respectively. Therefore, the shading time at different locations of the PV panels showed that under panels > behind panels > before panels > CK (0 min).

### 2.4 Measurement of growth indicators

A tape measure was used to determine the plant height of *A. adsurgens*, from the base of the stem to the distance between leaves in their upright position. Leaf length and width were measured using an electronic caliper with a precision of 0.01 mm. Leaf thickness was determined by measuring the combined thickness of the front, middle, and rear sections of the leaf, and the average value was calculated, excluding the central vein. The leaf was placed on a black cardboard with a known fixed area (used as a reference) and photographed from a fixed height. Photoshop software was used to process the image and calculate the location of a single leaf. The scanned leaves were then dried in an oven at 85°C until constant weight was achieved, and the dry weight of the leaf was determined using a balance with a precision of 0.001 g. The leaf area was calculated using the following formula:


(1) $$S_{single\_leaf}=\frac{N_{leaf}}{N_{reference}}\times S_{reference}$$

(2) $$SLA=\frac{S_{Single\_leaf}}{W_{Single\_leaf\;}}$$

where *S_single___leaf_* represents the area of a single leaf (cm^2^), *N_leaf_* is the number of pixels that correspond to the leaf area in the image, *N_reference_* is the number of pixels corresponding to the reference object in the same image. *S_reference_* is the area of the reference object (cm^2^). *SLA* represents the specific leaf area (cm²/g), and *W_single_leaf_* is the dry weight of a single leaf (g).

### 2.5 Measurement of physiological indicators

#### 2.5.1 Photosynthetic pigment determination

Chlorophyll (Chl) was determined by acetone extraction method. Briefly, 0.1 g of fresh leaves were cut into small pieces and mixed with quartz sand, calcium carbonate powder, and an appropriate amount of 80% acetone (a mixture of acetone and distilled water in a ratio of 4:1) to extract Chl adequately. The homogenate was then poured into a centrifuge tube to remove suspended solids and impurities. The supernatant was diluted, and the absorbance was measured at 646 and 663 nm using a spectrophotometer. Absorbance values were calculated using the following equations (Li, 2000):


(3) $$\text{Chl a}=\frac{\left[\left(12.21A_{663}-2.81A_{646}\right)\right]\times \text{V}\times \text{N}}{\text{W}\times 1000}$$

(4) $$\text{Chl b}=\frac{\left[\left(20.13A_{646}-5.03A_{663}\right)\right]\times \text{V}\times \text{N}}{\text{W}\times 1000}$$

(5) Chl=Chl a+Chl b

where, *A_663_* and *A_646_* are the absorbance values, V is the volume of the extract (ml), W is the number of grams of sample weighed (g), and N is the dilution.

#### 2.5.2 Determination of osmotic adjustment substances

The soluble sugar (SS) content was determined using the anthrone method (Seifter and Dayton,1950). The (soluble protein) SP content was determined by G250 Coomassie brilliant blue method (Bradford, 1976). One gram of frozen sample was ground in the precooling bowl with 1.5 ml of 80% ethanol (adding a little quartz sand), and the volume was fixed with 80% ethanol solution to 5 ml. The extract was transferred to a test tube at 80°C for 20 min. Then, the extract was filtered twice through filter paper with activated carbon. The filtrate was placed in a test tube with 0.2× the weight of zeolite and oscillated for 5 min. The supernatant was centrifuged at 4°C for 10 min at 5,000 × *g* and determined by acid ninhydrin colorimetry.

#### 2.5.3 Malondialdehyde (MDA) assaying

Malondialdehyde (MDA) was determined using the thiobarbituric acid method (Bates et al., 1973; Girotti, 1990). A 0.5-g sample of ground leaf tissue was mixed with 5 ml of 5% trichloroacetic acid. The mixture was centrifuged twice, first at 4,000 r/min and then at 3,000 r/min, to obtain the supernatant. Of the supernatant, 2 ml was taken and mixed with 2 ml of 0.67% thiobarbituric acid. The solution was boiled for 30 min, cooled, and centrifuged again. The absorbance of the solution was measured using a spectrophotometer at 450, 532, and 600 nm, and the MDA content was calculated using the following formula (Gao, 2006):


(6) $$\text{C}\left(\text{MDA}\right)=\frac{\left[6.452\times \left({\text{A}}_{532}-{\text{A}}_{600}\right)-0.56\times {\text{A}}_{450}\right]\times \text{V}}{\text{W}\times 1000}$$

where V represents the total volume of the extract (ml), and W represents the sample fresh weight (g).

#### 2.5.4 Determination of enzyme activity

Superoxide dismutase (SOD) was determined using the nitroblue tetrazolium method (Li, 2000). Peroxidase (POD) was determined using the ultraviolet absorption method (Dhindsa and Matowe, 1981). Approximately 0.1 g of the sample was weighed, and 1 ml of extraction buffer was added. The mixture was centrifuged at 8,000 × *g* for 10 min at 4°C, and the supernatant was collected. The absorbance of the solutions was measured at 560 nm and 240 nm using kits provided by Solarbio (Beijing, China).

#### 2.5.5 Measurement of mineral elements

Mineral elements of stems and leaves were determined using the H_2_SO_4_–H_2_O_2_ digestion method (Li, 2000). One gram of sample of ground stem and leaf tissue was digested with concentrated sulfuric acid and hydrogen peroxide to break down organic matter and release nutrients. After digestion, the N (nitrogen) content was measured by the Kjeldahl method, and the P (phosphorus) content was measured by the vanadomolybdate yellow colorimetric method. Finally, the CP (crude protein) content and N:P ratio were calculated to evaluate the nutrient status and balance of the sample.

### 2.6 Statistical analysis

Before analyses, the Shapiro–Wilk normality test and Levene’s test were used to test the normality and homogeneity of variance of the variables, respectively. If the assumptions were met, one-way analysis of variance (ANOVA), LSD multiple comparison tests, and correlation analyses were used for multiple comparisons. All statistics were performed using SPSS software (v.20.0, SPSS Inc., Chicago, USA). Significant differences were indicated with letter markers. A membership function method was used to comprehensively evaluate the *A. adsurgens* plants at different locations. All graphical representations were generated using Origin 2024 software (OriginLab, USA).

## 3 Results

### 3.1 Growth characteristics of *A. adsurgens* at different locations in PV station

As shown in **Table 1**, during the growing season (June–August), the plant height, leaf length, and leaf width of *A. adsurgens* growing before, behind, and under the fixed PV panels were greater than those of the CK. The growth was most pronounced in the under panels, with increases of 49.67%, 58.43%, and 21.84%, respectively, by the end of the observation period. In contrast, leaf thickness followed the CK > before panels > behind panels > under panels. Moreover, the SLA was highest in under panels, reaching a peak of 221.86 cm²·g⁻¹ in August, indicating the most muscular photosynthetic capacity in this area. In summary, the *A. adsurgens* plants surrounding the PV panels adapted to the shaded environment by adjusting their height and leaf morphology to enhance light capture compensating for reduced photosynthesis. On the other hand, the CK adapted by developing shorter, thicker, and smaller leaves to improve water retention and reduce transpiration. These adaptive changes are closely related to microclimate variations and promote the growth and development of *A. adsurgens* around the PV panels.

**Table 1:** Changes in growth characteristics in different locations of PV panels with *A. adsurgens*.

Index	Time	Before panels	Behind panels	Under panels	CK
Plant height/cm	June	24.10 ± 0.95b	23.13 ± 0.71b	27.67 ± 1.14a	21.07 ± 1.28c
	July	40.82 ± 4.51ab	33.15 ± 0.41bc	48.68 ± 2. 89a	25.97 ± 1.42c
	August	52.57 ± 3.20b	43.56 ± 5.31c	61.33 ± 3.30 a	30.87 ± 2.29d
Leaf length/cm	June	2.21 ± 0.08a	2.20 ± 0.08a	2.22 ± 0.15a	1.98 ± 0.11b
	July	1.89 ± 0.04b	2.28 ± 0.15a	2.36 ± 0.25a	1.83 ± 0.06b
	August	1.99 ± 0.08b	2.26 ± 0.10b	2.55 ± 0.18a	1.06 ± 0.05c
Leaf Width/cm	June	0.69 ± 0.03b	0.91 ± 0.06a	0.64 ± 0.05b	0.62 ± 0.04b
	July	0.68 ± 0.02b	0.88 ± 0.05a	0.84 ± 0.06a	0.63 ± 0.04b
	August	0.71 ± 0.05b	0.95 ± 0.03a	0.87 ± 0.05a	0.68 ± 0.03b
Leaf thickness/mm	June	0.24 ± 0.02a	0.19 ± 0.03b	0.14 ± 0.02c	0.25 ± 0.021a
	July	0.21 ± 0.01a	0.16 ± 0.02b	0.14 ± 0.03c	0.22 ± 0.02a
	August	0.26 ± 0.022a	0.17 ± 0.014b	0.11 ± 0.02b	0.28 ± 0.03a
Single leaf area/cm^2^	June	0.78 ± 0.03b	1.27 ± 0.13a	0.78 ± 0.09b	1.44 ± 0.21a
	July	0.89 ± 0.14b	1.35 ± 0.08a	1.43 ± 0.146a	1.08 ± 0.09b
	August	0.81 ± 0.08b	0.88 ± 0.01b	1.38 ± 0.18a	0.69 ± 0.05b
Single leaf dry weight/mg	June	6.62 ± 0.83b	7.17 ± 0.13b	5.44 ± 0.49c	8.77 ± 0.21a
	July	6.64 ± 0.14a	6.85 ± 0.38a	6.99 ± 0.16a	6.17 ± 0.19b
	August	5.88 ± 0.28b	5.91 ± 0.31b	6.22 ± 0.18b	7.32 ± 0.25a
Specific leaf area/(cm^2^·g^−1^)	June	117.82 ± 15.42c	177.12 ± 18.98a	143.38 ± 21.29b	164.20 ± 6.55a
	July	134.03 ± 12.71b	197.08 ± 6.73a	204.57 ± 10.37a	175.04 ± 7.74ab
	August	137.75 ± 18.93b	148.90 ± 15.11b	221.86 ± 19.06a	94.26 ± 12.19c

Different lowercase letters in the same line indicate significant difference at p < 0. 05 level.

### 3.2 Nutrient content of stems and leaves from *A. adsurgens* at different locations in PV Station

**Figure 3:**
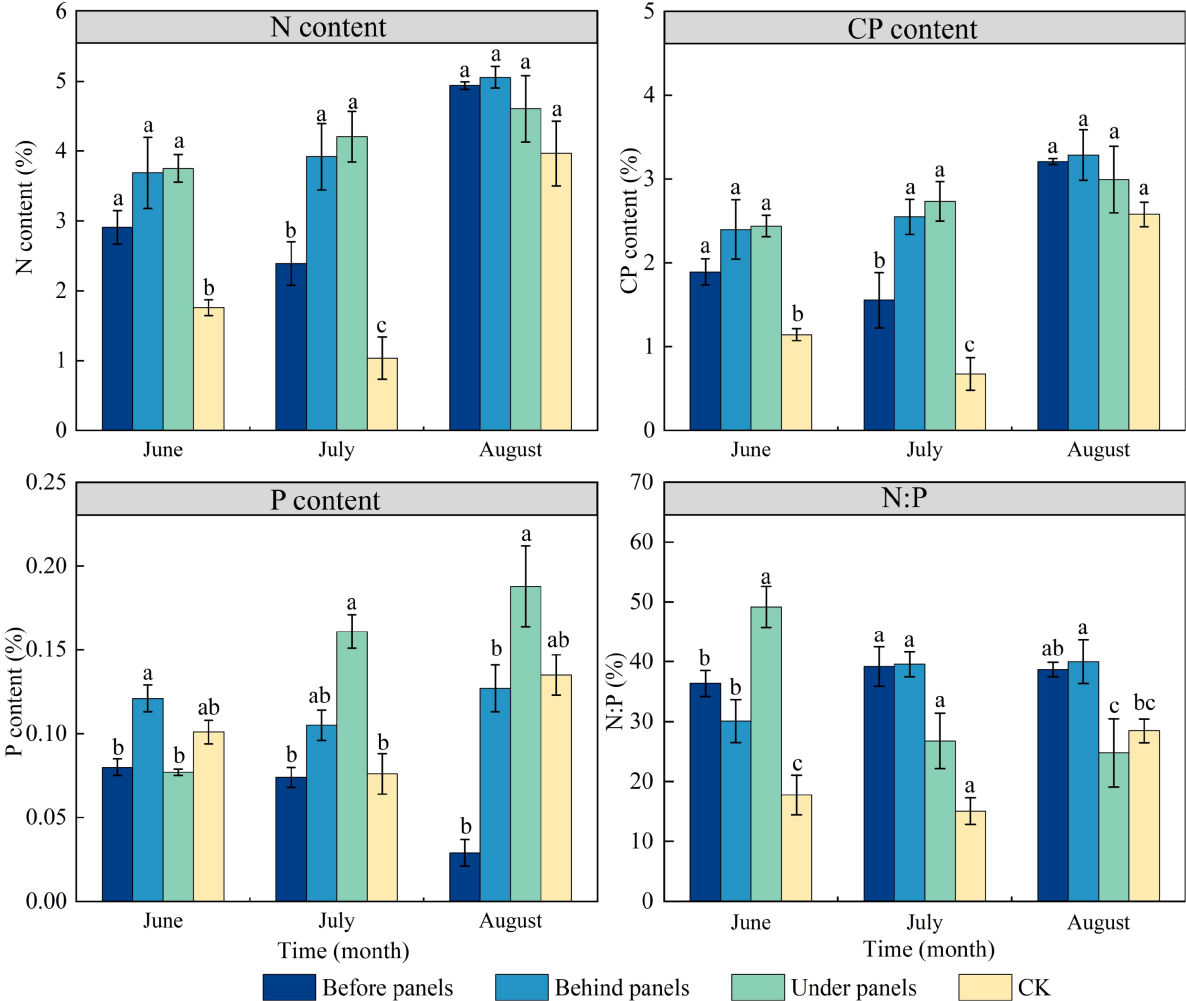
Changes in N (Nitrogen), CP (Crude Protein), P (Phosphorus), and N:P content in different locations of PV panels in *A. adsurgens.* Different lowercase letters indicate significant differences between treatments at the same time in the *p < 0.05* level.

During the growing season, the N and CP content of *A. adsurgens* located before, behind, and under PV panels were significantly higher than those in the CK, with increases of 51.40%, 87.30%, and 85.81%, respectively (**Figure 3**). In June, the P content was highest in plants growing under panels. However, in July and August, the under panels had the highest P content, reaching 0.16% and 0.19%, respectively. Throughout the growing season, the N:P of *A. adsurgens* exceeded 14 indicating that its growth was not limited by N, and the presence of PV panels did not negatively impact nitrogen accumulation. The N:P in the before, behind, and under panels’ plants were all greater than 16, suggesting that *A. adsurgens* growth in these locations was limited by P. In July, the N:P in the CK ranged between 14 and 16, which showed that its growth was co-limited by both N and P. Thus, planting *A. adsurgens* around PV panels can prevent N limitation and improve its N absorption capacity.

### 3.3 Effects of PV panels on the physiological characteristics of *A. adsurgens*

#### 3.3.1 Chlorophyll

Chl is a key substance in plants for absorbing and transferring light energy during photosynthesis (Hauenstein et al., 2016). The Chl content of *A. adsurgens* at the same location gradually increased over time, with significant differences (*p < 0.05*) observed between the areas around the PV panels and the CK (**Table 2**). Each month, the Chl content followed the trend: under panels > behind panels > before panels > CK. By August, the Chl a and Chl b contents under the panels had increased to 1.37 and 0.35 mg·g⁻¹, respectively, with the total Chl content reaching 1.72 mg·g⁻¹. Throughout the study, the Chl a/b ratio in all locations near the PV panels remained consistently lower than that of the CK indicating that the shading effect of the panels prompted *A. adsurgens* to reduce the Chl a/b ratio, thereby optimizing its light-harvesting efficiency and making it better adapt to low-light conditions.

**Table 2:** Changes in chlorophyll a (Chl a), chlorophyll b (Chl b), chlorophyll (Chl), and chlorophyll a/b (Chl a/b) of *A. adsurgens* in different PV panel positions during the growing season.

Index	Time	Before panels	Behind panels	Under panels	CK
Chl a (mg·g^−1^)	June	0.81 ± 0.05b	1.06 ± 0.06a	1.12 ± 0.06a	0.66 ± 0.07b
	July	1.10 ± 0.05ab	0.98 ± 0.04bc	1.17 ± 0.03a	0.87 ± 0.03c
	August	1.27 ± 0.12a	1.36 ± 0.09a	1.37 ± 0.05 a	0.88 ± 0.08b
Chl b(mg·g^−1^)	June	0.20 ± 0.01bc	0.25 ± 0.01ab	0.29 ± 0.02a	0.15 ± 0.02c
	July	0.28 ± 0.02ab	0.26 ± 0.01ab	0.34 ± 0.01a	0.23 ± 0.03b
	August	0.32 ± 0.05a	0.31 ± 0.02a	0.35 ± 0.01a	0.16 ± 0.02b
Chl (mg·g^−1^)	June	1.01 ± 0.05bc	1.31 ± 0.07ab	1.41 ± 0.08a	0.81 ± 0.09c
	July	1.38 ± 0.07ab	1.25 ± 0.04bc	1.51 ± 0.03a	1.10 ± 0.06c
	August	1.59 ± 0.17a	1.67 ± 0.10a	1.72 ± 0.05a	1.04 ± 0.10b
Chl a/b	June	4.05 ± 0.15a	4.24 ± 0.03a	3.86 ± 0.09b	4.40 ± 0.21a
	July	3.92 ± 0.14a	3.76 ± 0.08a	3.44 ± 0.04a	3.78 ± 0.48a
	August	3.96 ± 0.22b	4.39 ± 0.09b	3.91 ± 0.16b	5.50 ± 0.26a

Different lowercase letters in the same line indicate significant difference at p < 0. 05 level.

#### 3.3.2 Osmotic adjustment substances

**Figure 4:**
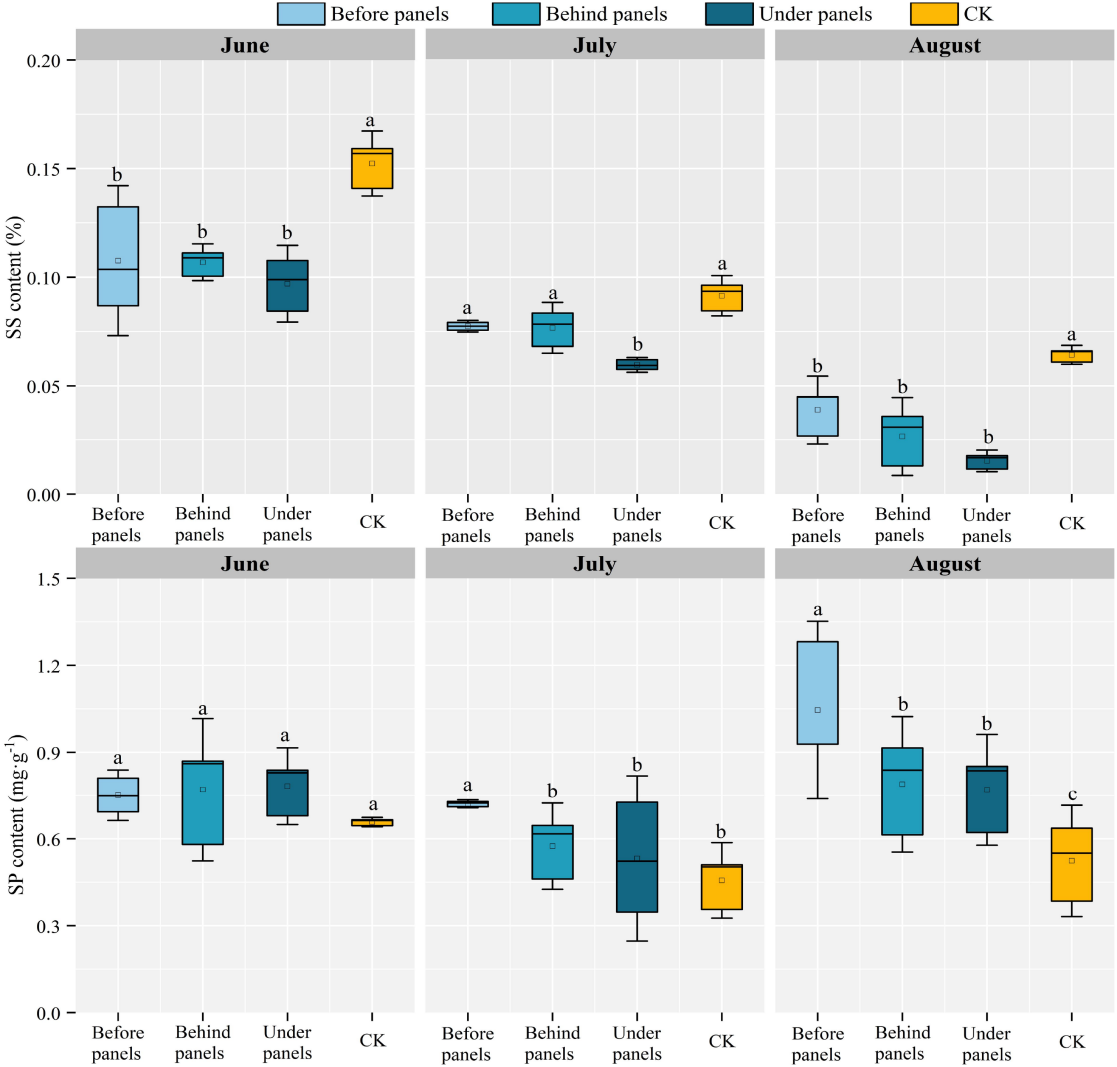
Variations in soluble sugar (SS) content and soluble protein (SP) content of *A. adsurgens* in different locations of the PV panels. Different lowercase letters indicate significant differences between treatments at the same time in the *p < 0.05* level.

SS and SP are important osmotic regulators helping retain water by reducing plant water potential (Zhang et al., 2022). The SS content of *A. adsurgens* in different locations in the fixed PV station decreased over time, with the largest decline observed under panels, which decreased by 84.54% (**Figure 4**). Within the same month, the SS content showed a clear gradient: CK > before panels > behind panels > under panels. In June, the SS content at the CK location reached its highest at 0.152%, while the content under panels was the lowest in August, only 0.015%. The SP content of *A. adsurgens* in different locations (before, behind, and under the panels) first decreased and then increased over time reaching its lowest levels in July at 0.721, 0.575, and 0.53 mg·g^−1^, respectively. Significant differences in SP content were observed at different growth stages across the various locations (*p < 0.05*). In July, the SP content decreased most significantly in the behind and under panel areas, with reductions of 25.37% and 31.93%, respectively; in August, the SP content before the panels was the highest, reaching 1.05%.

#### 3.3.3 Malondialdehyde

**Figure 5:**
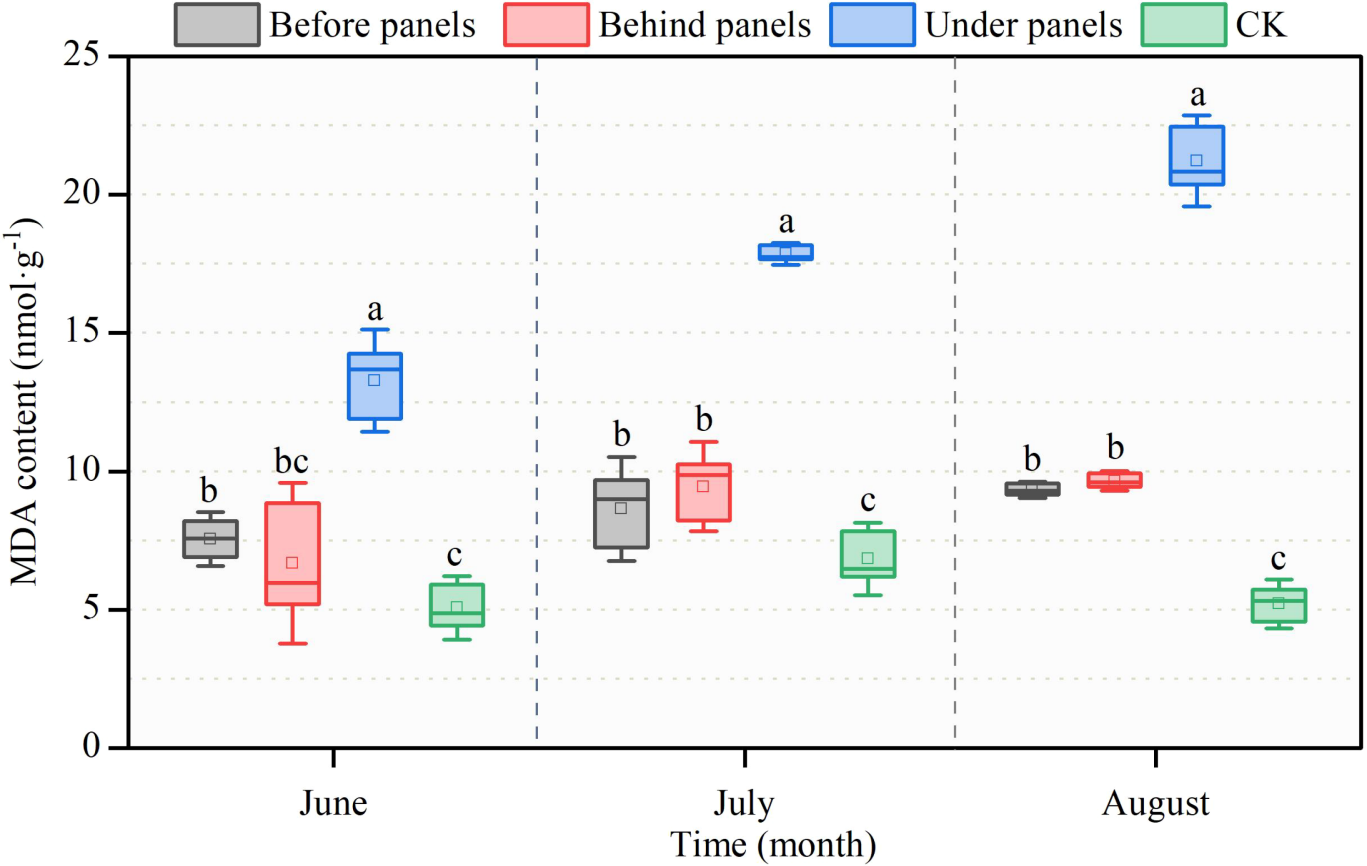
Changes in malondialdehyde (MDA) content in different locations of PV panels from June to August. Different lowercase letters indicate significant differences between treatments at the same time in the *p < 0.05* level.

Changes in malondialdehyde (MDA) content are closely related to the light conditions and stress levels experienced by the plants (Kong et al., 2016). As the growing season progressed, MDA content in *A. adsurgens* increased in all locations near the PV panels, with the highest value of 21.21 nmol·g^−1^ observed in August under panels (**Figure 5**). This was the highest level recorded during the observation period. In contrast, the MDA content at the CK location followed a trend of first increasing and then decreasing peaking in July at 6.84 nmol·g^−1^. Throughout the growing season, the SP content at the CK location was consistently the lowest showing reductions of 61.78%, 61.70%, and 75.43% compared to the highest values observed under panels.

#### 3.3.4 Enzyme activity

**Figure 6:**
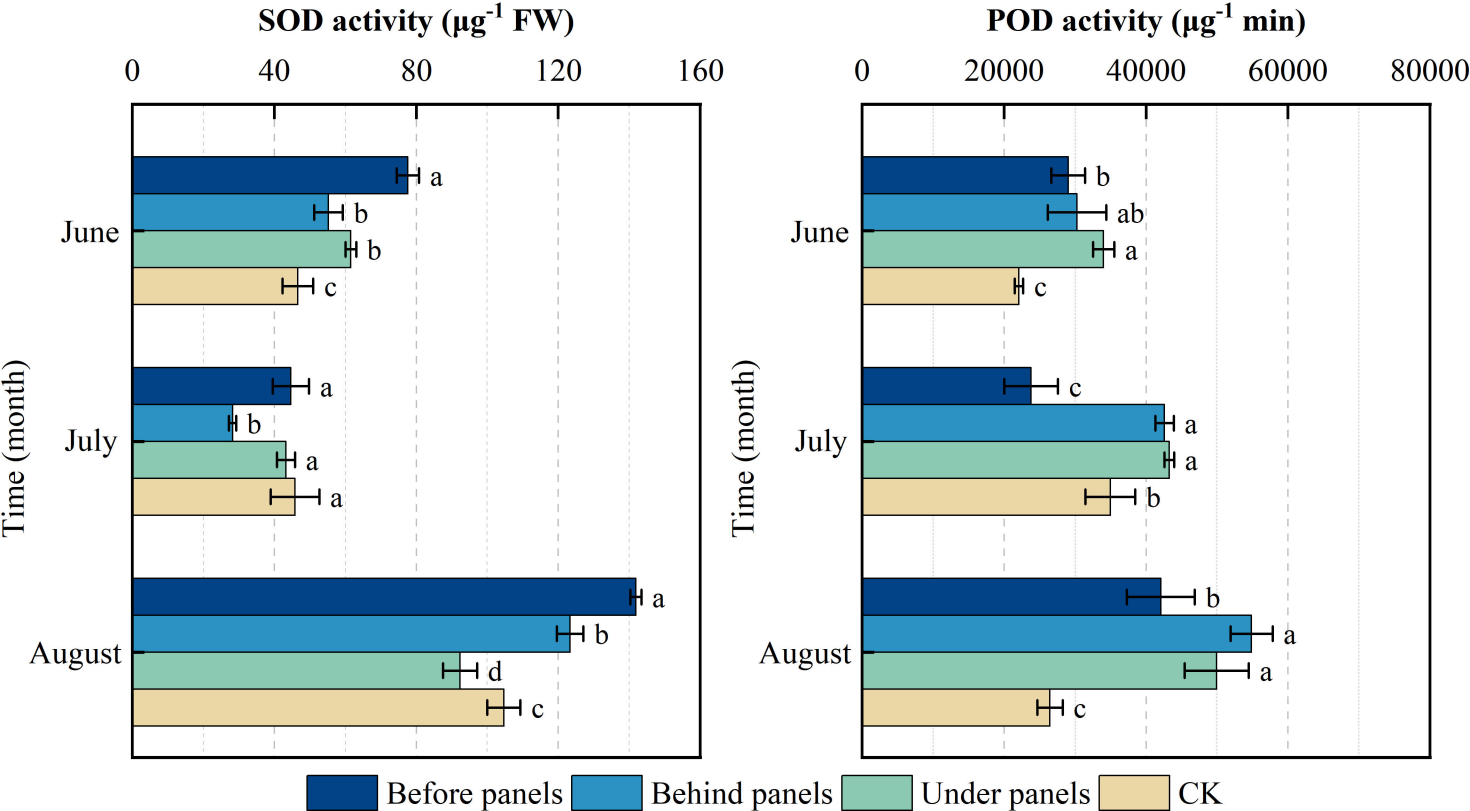
Changes in superoxide dismutase (SOD) and peroxidase (POD) in different locations of photovoltaic panels from June to August. Different lowercase letters indicate significant differences between treatments at the same time in the *p < 0.05* level.

**Figure 6** illustrates the critical role of SOD and POD activities in helping plants resist environmental stress. Under the influence of PV panels, the activities of POD and SOD in *A. adsurgens* exhibited significant differences across different locations (*p < 0.05*). Over time, the SOD activity in plants showed an initial decrease followed by an increase, while POD activity consistently rose, with both enzymes reaching their peak activities in August. Specifically, SOD was the highest with 141.86 µg^−1^ FW before panels, whereas POD activity peaked at 54,863.33 µg^−1^·min under panels. The rise in summer temperatures and light intensity likely increased the production of superoxide anion radicals and hydrogen peroxide in the plants, stimulating heightened enzyme activity. In the same month, POD activity behind and under panels was significantly higher than that before panels and the CK (*p < 0.05*). Additionally, SOD activity before panels was significantly higher than that in the CK (*p < 0.05*). These results suggest that *A. adsurgens* plants rely on increased POD and SOD activities to effectively cope with the shading-induced stress from the PV panels enabling them to maintain normal physiological functions.

### 3.4 Comprehensive analysis of the growth and physiological status of *A. adsurgens* in different locations in PV Station

**Figure 7:**
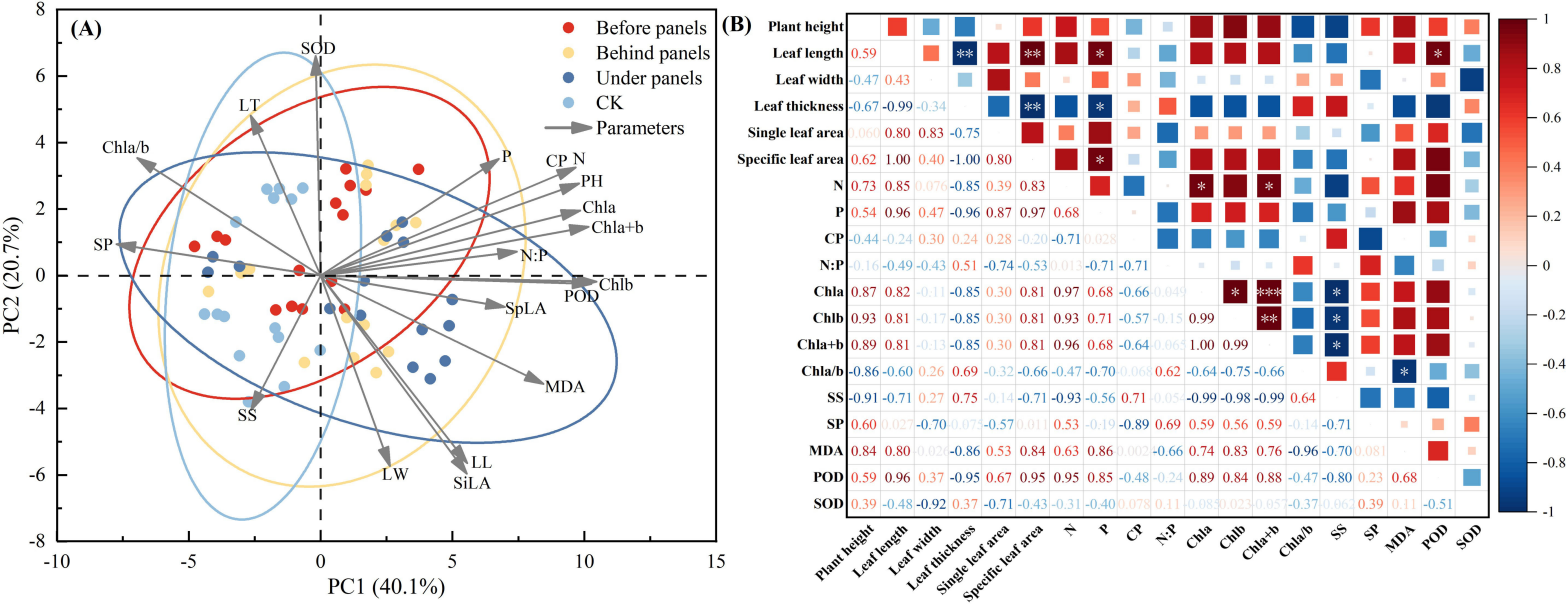
Comprehensive analysis of growth and physiological indices of *A. adsurgens.* **(A)** Principal component analysis. **(B)** Correlation analysis.

Principal component analysis (PCA) showed that within the 95% confidence interval, the influence of different locations around the PV panels on the growth and physiological indices of *A. adsurgens* exhibited significant differences. PC1 and PC2 explained 60.8% of the variation in plant growth and physiology highlighting the critical impact of PV panel interference on growth and physiological characteristics (**Figure 7A**). Specifically, leaf length, SLA, P content, and POD activity were positively correlated (*p < 0.05*) (**Figure 7**). Conversely, leaf thickness was negatively correlated with SLA and P content (*p < 0.05)* indicating that *A. adsurgens* adopts adaptive strategies to adjust to different light conditions. In addition, SS were negatively correlated with Chl content (*p < 0.05*), while Chl a/b was negatively correlated MDA content (*p < 0.05*) (**Figure 7B**). Finally, a comprehensive evaluation of the growth and physiological status of *A. adsurgens* was conducted using a membership function analysis of 19 physiological indicators. The average membership function value for each indicator at a given location was used as a quantitative measure of plant growth, with higher values indicating better growth conditions. As shown in **Table 3**, the ranked growth status of *A. adsurgens* in different locations around the PV panels is as follows: under panels > before panels > behind panels > CK.

**Table 3:** Membership function analysis of *A. adsurgens* in different locations in PV panels.

	Before panels	Behind panels	Under panels	CK
Plant height	0.530	0.481	0.508	0.468
Leaf length	0.542	0.337	0.644	0.523
Leaf width	0.478	0.501	0.594	0.460
Leaf thickness	0.570	0.419	0.444	0.406
Single leaf area	0.546	0.429	0.535	0.536
Specific leaf area	0.549	0.509	0.489	0.485
N	0.504	0.469	0.510	0.415
P	0.472	0.576	0.586	0.473
Chl a	0.491	0.501	0.481	0.447
Chl b	0.551	0.498	0.465	0.471
Chl a + b	0.509	0.505	0.513	0.460
Chl a/b	0.479	0.470	0.583	0.492
SS	0.731	0.582	0.582	0.620
SP	0.466	0.617	0.593	0.650
MDA	0.555	0.481	0.457	0.457
SOD	0.473	0.456	0.509	0.525
POD	0.503	0.519	0.502	0.424
Average value	0.526	0.491	0.529	0.489
Tolerance of plants	2	3	1	4

## 4 Discussion

The construction of PV power plants has significantly altered local microclimatic conditions directly impacting key ecological factors essential for the growth of *A. adsurgens*, including solar radiation, temperature, humidity, and moisture (Chang et al., 2020; Zhang et al., 2023). Experimental results reveal significant variation in sunshine duration within the PV plant ranked in shading as follows: under panels > behind panels > before panels > CK. This disparity arises from the shading effect of the PV panels, which reduces solar radiation at various locations, resulting in distinct microclimates. These microclimates differ significantly from surrounding areas in temperature, humidity, and wind conditions. For example, temperature under the panels was consistently lower than that in the open areas, with soil temperatures in the 0- to 40-cm layer dropping by 16%–17% compared to unshaded CK (Yue et al., 2021a). Humidity levels were higher under panels, as the shading effect reduced evaporation rates, creating a more humid environment compared to areas with full exposure to sunlight. Wind conditions were also altered; the PV panels obstructed wind flow leading to reduced wind speeds under and behind the panels, while areas before panels experienced higher wind velocities because of less obstruction. Collectively, these shifts in temperature, humidity, and wind conditions have complex effects on the growth and reproduction of local ecosystems influencing the development of *A. adsurgens* in the PV station area. Sansaniwal et al. (2018) found that solar radiation within the PV station was 9.05% lower than unshaded control sites, further reinforcing the impact of PV panel configuration on the local microclimate.

### 4.1 Effects of PV arrays on the growth morphology of *A. adsurgens*

The root, stem, and leaves are critical organs for photosynthesis and respiration, and environmental conditions are primary factors influencing plant growth (Desalme et al., 2011; Yin et al., 2021; Lv et al., 2023). This study demonstrates that the height, leaf length, and leaf width of *A. adsurgens* near the PV panels were 1.10 to 1.99, 1.01 to 2.40, and 1.03 to 1.47 times greater, respectively, compared to those of the control group (**Table 1**). This suggests that the environmental modifications induced by the PV arrays facilitated plant growth. Notably, *A. adsurgens* exhibited the most pronounced growth in under panels. By reducing sunlight exposure and solar radiation, the PV panels altered the red to far-red light ratio affecting the plant’s photoreceptor response and promoting increased plant height and leaf size for more efficient light capture. Furthermore, growth disparities between the before and behind the panels suggest that, although the area behind panels experiences longer shading periods, the light and microclimate conditions before panels are more conducive to plant growth. These findings are consistent with those of Yue et al. (2021a), who reported that PV panels provide a cooling and humidifying effect in summer, particularly in arid regions, thereby enhancing the vegetation growth environment. The growth characteristics of *A. adsurgens* in this study highlight its strong adaptability to heterogeneous light conditions.

### 4.2 Effects of PV arrays on the physiological functions of *A. adsurgens*

N and P are indispensable nutrients for plant growth, while CP is an important indicator of plant quality (Kumar et al., 2021). This study revealed that the N and CP contents of *A. adsurgens* around the PV panels were higher than those in CK, and the P content under panels was significantly increased (**Figure 3**). This was possible because increased blue-violet light prompted the conversion of carbon metabolism to N metabolism, while shade reduced photosynthetic products, thereby promoting N accumulation. The N:P is often used as a measure of plant growth limitation. Koerselman and Meuleman (1996) suggested that plant growth and development are mainly limited by N when the N:P is less than 14, P when the ratio is greater than 16, and both when the ratio is between 14 and 16. The N:P around the PV panels were all greater than 16, which shows that planting *A. adsurgens* in PV plants can effectively avoid the N limitation on their growth. Additionally, the shading effect of the PV panels increased Chl b content in *A. adsurgens* enhancing its ability to utilize light energy in low-light conditions. The increase in Chl content was also linked to soil moisture (Liu et al., 2018) aligning with this study’s finding that shading duration under the PV panels was the longest, and Chl content was higher than that in CK (**Table 2**). During the summer months (June to August), temperatures in the study area increased peaking in July. Changes in soil moisture often lag behind temperature fluctuations, and plants are more sensitive to soil moisture than temperature (Sun et al., 2018). As a result, *A. adsurgens* increased its osmotic adjustment capability in late August to enhance water retention in response to declining soil moisture. Moreover, the SS and SP contents of *A. adsurgens* near the PV panels were higher than those in CK indicating that the plant’s accumulated intracellular solutes improved water retention and adapted to reduced soil moisture (**Figure 4**). Elevated MDA levels indicated that plants under the panels may experience environmental stress (**Figure 5**). Still, increased SOD and POD activities reflected the strong antioxidant capacity of *A. adsurgens* further confirming its resilience to the conditions under the PV panels (**Figure 6**).

### 4.3 Application potential and optimization strategies for *A. adsurgens* in ecological restoration within PV power stations

This study underscores the ecological potential of *A. adsurgens* in different locations within a PV power station and concluded that, due to the shading effect from PV panels, *A. adsurgens* shows the highest tolerance under panels, followed by the areas before and behind panels, with the CK showing the lowest tolerance (**Table 3**). Based on this, the following optimization strategies were recommended to maximize the application of plants in ecological restoration within PV stations: 1. Optimization of the ecological layout before panels: Given the favorable light conditions in this area, it is recommended to plant low-growing, drought-resistant, and wind erosion-resistant species to minimize shading impacts on PV panel efficiency while enhancing the station’s wind erosion control. 2. Enhance fertilization management: During the growth period of *A. adsurgens*, increase the application of P fertilizer and adjust the N:P ratio to alleviate P limitation and improve plant growth efficiency. Fertilizer application should be adjusted based on soil conditions, with careful attention to avoid overfertilization, which could lead to nutrient imbalances or soil salinization. Water management should also be coordinated to prevent fertilizer runoff. 3. Regular monitoring and dynamic adjustments: It is recommended to deploy environmental monitoring sensors to track soil moisture, temperature, and other relevant data in real time and periodically assess plant growth conditions. Based on the monitoring results, adjustments should be made to irrigation volumes, fertilization strategies, and planting density to ensure the healthy growth of vegetation. This optimization measures fully exploit the significant ecological potential of *A. adsurgens* in PV power stations effectively promoting the harmonious coexistence of PV energy generation and ecological environmental protection.

## 5 Conclusion

The *A. adsurgens* growing around PV panels showed significant growth advantage, with greater plant height, leaf length, leaf width, and nutrient content in stems and leaves compared to those of CK. This indicates its strong adaptability to the ecological environment of PV power stations. During the peak growing season, *A. adsurgens* successfully adapted to the environmental conditions around the PV panels by increased Chl content and modulating the levels of SS and SP. Furthermore, the activities of SOD and POD were notably higher at all positions within the PV station compared to those of CK, which effectively resisted oxidative stress in this environment. Through membership function analysis, the tolerance ranking of *A. adsurgen* in different positions was under panels > before panels > behind panels > CK reflecting the positive influence of the PV panels on its growth. This study provides a scientific basis for demonstrating the ecological potential of PV power plants in desert areas and offers practical guidance for vegetation restoration and ecological construction around PV power plants. Future studies could further explore the adaptive mechanisms of different plant species within PV power plants and how to achieve broader ecological benefits by better integrating PV power generation with ecological conservation.

## Data Availability

The original contributions presented in the study are included in the article/supplementary material. Further inquiries can be directed to the corresponding author.
